# Supplementary material for: The interactome of intact mitochondria by cross-linking mass spectrometry provides evidence for coexisting respiratory supercomplexes
Source: Mol Cell Proteomics. 2017 Dec 8;17(2):216–32. doi: 10.1074/mcp.RA117.000470 (PMC5795388; doi:10.1074/mcp.RA117.000470)
Supplement: Supplemental Data [file supp_RA117.000470_133922_0_supp_23978_fzffwf.zip › spectra_annotation/mito_spectra_annotation/87-1-2-1-1-1.pdf]

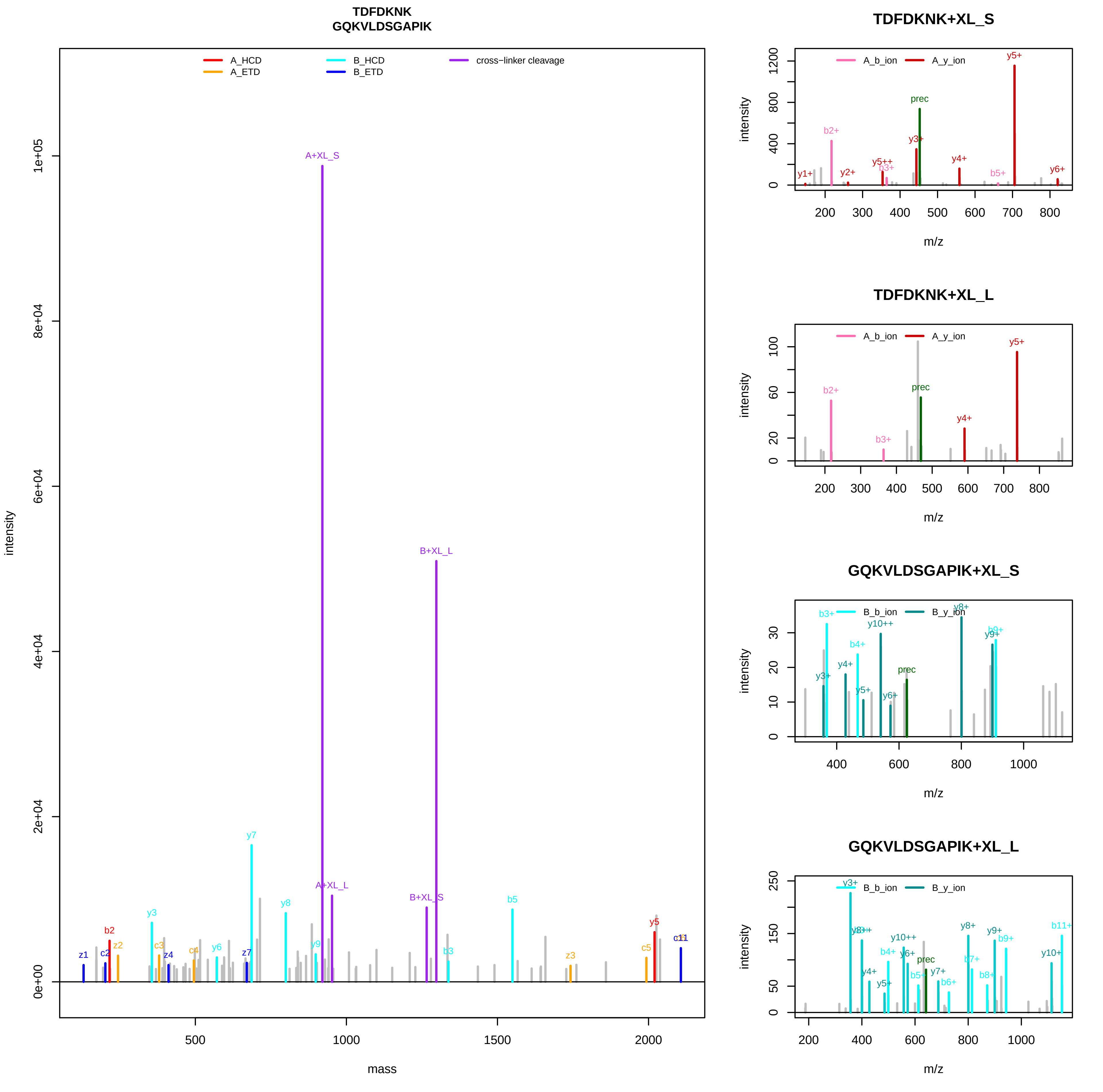
TDFDKNK
GQKVLDSGAPIK
A_HCD
A_ETD
B_HCD
B_ETD
cross−linker cleavage
A+XL_S
B+XL_L
A+XL_L
B+XL_S
z1
c2
b2
z2
y3
c3
z4
c4
y6
z7
y7
y8
y9
b3
b5
z3
c5
y5
c11
intensity
mass
0e+00
2e+04
4e+04
6e+04
8e+04
1e+05
500
1000
1500
2000
TDFDKNK+XL_S
A_b_ion
A_y_ion
y1+
b2+
y2+
y5++
b3+
y3+
prec
y4+
b5+
y5+
y6+
m/z
TDFDKNK+XL_L
b2+
b3+
prec
y4+
y5+
GQKVLDSGAPIK+XL_S
B_b_ion
B_y_ion
b3+
y3+
y4+
b4+
y5+
y10++
y6+
prec
y8+
y9+
b9+
GQKVLDSGAPIK+XL_L
y3+
y8++
y4+
b4+
y5+
y10++
y6+
b5+
prec
y7+
b6+
y8+
b7+
b8+
y9+
b9+
y10+
b11+
